# Carbozotate of Ammonia

**Published:** 1874-10

**Authors:** 


Carbozotate of Ammonia.—Dr. W. C. Person, Air Mount, Miss., in a letter to the July number of the *St. Louis Medical and Surgical Journal,* says: The objections to quinine are numerous. * * * The inducement to adulterate is too great. The quantity necessary to prevent a paroxysm of pernicious intermittent, in this section, would astonish the physicians who accept the teachings of the Dispensatory, in regard to quantity. * * * Repeated disappointments in its effects last summer induced me to experiment with carbozotate of ammonia. * * I have been using it constantly ever since in a great many cases requiring the use of an antiperiodic with more uniform success than I generally derived from quinine or arsenic either. Several of my cases seem to indicate that it arrests the paroxysms more completely than quinine. I have succeeded with it in cases of chronic quotidian ague of long standing, while quinia, arsenic, and the whole catalogue of tonics and alteratives, had been tested thoroughly. For ordinary chills, so prevalent in the Southern States, I have ceased using quinine entirely. In order to test the virtues of the drug more fully, I divided my stock of the salt with another physician, who reports perfect success. In one case, however, it produced free emesis and intense cephalalgia—the effects of an overdose, probably. It is rarely necessary to give more than one grain, twice daily, until I give six grains. I have never had occasion to push the remedy to the extent of producing discoloration of the skin. I have not tested its efficacy so thoroughly in remittents as I have in intermittents. But my observation, as far as it extends, proves it to be as effective in one form as the other. I have given it regardless of febrile excitement, with impunity, and with the happiest effect. The result of my experience is, that while quinine possesses so many valuable and varied therapeutical properties that it is not likely to be superceded by any other remedy, yet, carbozotate of ammonia possesses properties which render it preferable to quinine, in a great many instances, as an antiperiodic, and is, therefore, worthy of further trial, especially by Southern physicians.—*Virginia Medical Monthly.*

